# Supplementary material for: Volumetric Light-field Encryption at the Microscopic Scale
Source: Sci Rep. 2017 Jan 6;7:40113. doi: 10.1038/srep40113 (PMC5216341; doi:10.1038/srep40113)
Supplement: Supplementary Information [file srep40113-s1.pdf]

Supplementary Information for

Volumetric Light-field Encryption at the Microscopic Scale

Haoyu Li, Changliang Guo, Inbarasan Muniraj, Bryce C. Schroeder, John T. Sheridan and Shu Jia

Supplementary Figure 1	Light-field encryption using random amplitude masks
Supplementary Figure 2	Digitization for binary storage and read-out of the encrypted data using random amplitude masks
Supplementary Figure 3	Volumetric encryption and decryption of non-binary objects
Supplementary Note	Pixel size of the numerical model of light-field propagation and image formation

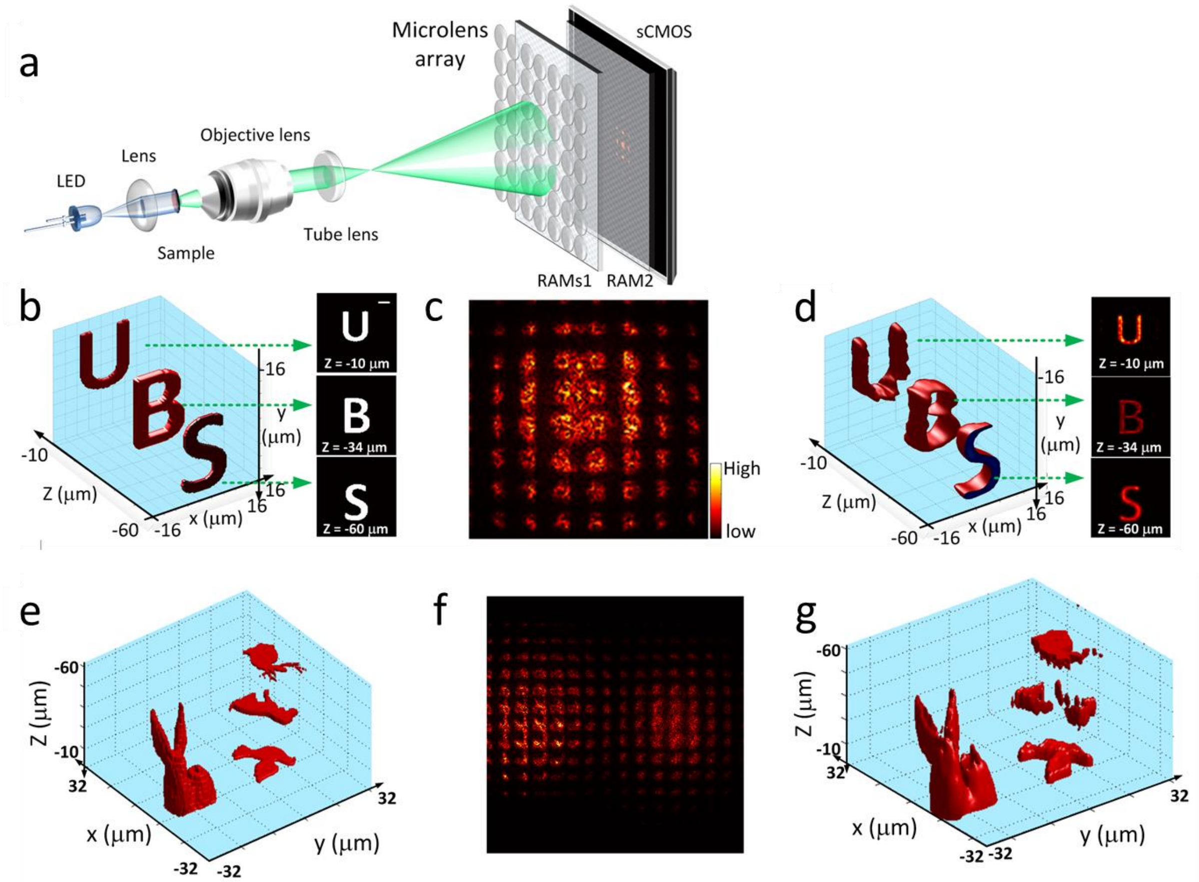

Supplementary Figure 1. Light-field encryption using random amplitude masks (RAMs). (a) Optical setup of the light-field encryption system. The object is imaged by the objective lens, and an intermediate image is formed by the tube lens. The microlens array is located at the intermediate image plane of the tube lens. An array of random amplitude masks (RAMs1) is located next to the microlens array. A camera sensor is placed at a distance of $f_{\mu lens}$ after the microlens array and RAMs1. The second random amplitude mask (RAM2) is located in front of the CCD camera. (b) Volumetric light-field encryption and decryption of three volumetric objects of the letters 'S', 'B' and 'U', located at -60 μm, -34 μm, and -10 μm along the axial (z) dimension, respectively. (c) The corresponding encrypted 128-pixel x 128-pixel 2D light-field image. Visual scrutiny has been largely suppressed. (d) Decrypted 3D volumetric information using the PSF key. (e) Optical encryption of multiplexed volumetric data, containing discrete and continuous volumetric data. (f) The encrypted 256-pixel x 256-pixel 2D light-field image. (g) Decryption and reconstruction of 3D volumetric information. Scale bar in (b), 4 μm.

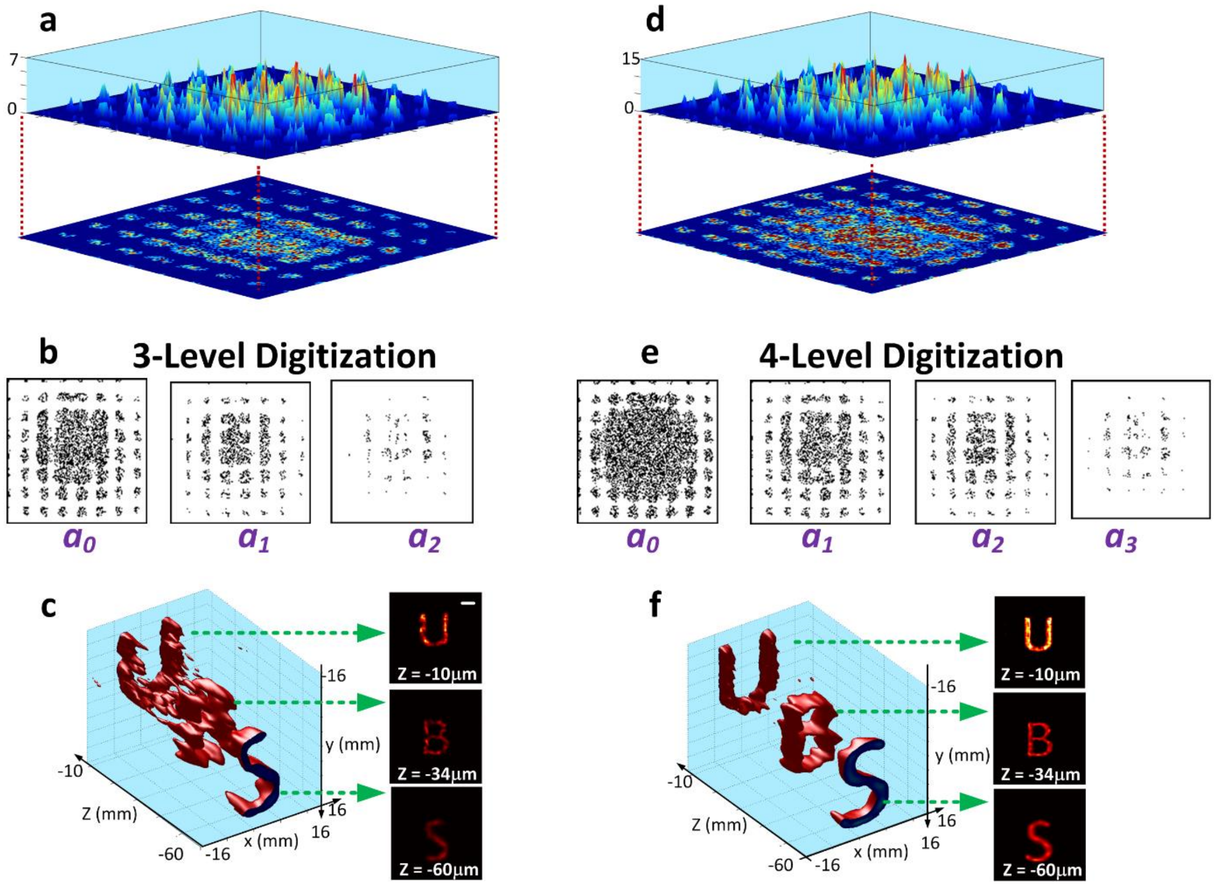

Supplementary Figure 2. Digitization for binary storage and read-out of the encrypted data using random amplitude masks (RAMs). (a) *N* = 3 level digitization of the encrypted 2D light-field image in Figure 2b. Pixel values of the light-field image were normalized to $2^N - 1 = 7$ and rounded to the closest integer values *P*, which can be expressed as $P = a_0 \cdot 2^0 + a_1 \cdot 2^1 + a_2 \cdot 2^2$, where $a_i = 0\ or\ 1\ (i = 0, 1, 2)$ is the pixel-value composite coefficient. (b) The respective images that represent the binary composites of the pixel values of the light-field image at $2^0 = 1$, $2^1 = 2$, and $2^2 = 4$, respectively. (c) Decrypted volumetric data using digitized images in (b), revealing the original volumetric information. (d) *N* = 4 level digitization of the same 2D light-field image. (e) Respective binary images. (f) Decrypted volumetric data using digitized images in (e), showing a high-quality reconstruction comparable to Supplementary Figure 1d using the full light-field information. Scale bar in (c), 4 μm.

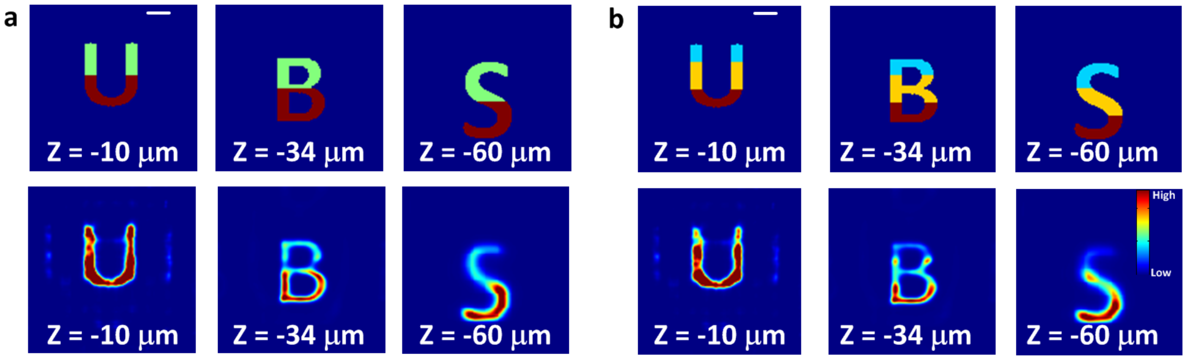

Supplementary Figure 3. Volumetric encryption and decryption of non-binary objects. (a) Top row, 3D volumetric objects spatially organized as in Figure 2. The amplitude of the objects is distributed across three levels (0-blue, 128-green, 255-red). Bottom row, decrypted 3D information following the same procedures as in Figure 2. The variations in amplitude can be visualized. (b) Top row, the amplitude of the objects is distributed across four levels (0-blue, 85-light blue, 170-yellow, 255-red). Bottom row, decrypted 3D volumetric information. The variations in amplitude can be visualized. Scale bars, 4 μm.

Supplementary Note - Pixel size of the numerical model of light-field propagation and image formation

A *Spectral Method* [1,2] model is adopted in Eq. 5 in the manuscript (rewritten below as Supplementary Eq. 1), considering that the free space propagation in z is a near-field Fresnel patterns calculation. This process is given by performing the Fourier transform on the input information followed by multiplying the spectrum chirp, and finally the inverse Fourier transform is taken on the resulting information in the Fourier space.

$$h(\mathbf{x},\mathbf{p}) = \mathcal{F}^{-1}\left\{\mathcal{F}[U_i(\mathbf{x},\mathbf{p})\Phi(\mathbf{x})R(\mathbf{x})] \times \exp\left[i2\pi\lambda f_{\mu lens}\sqrt{1-\left(f_x^2+f_y^2\right)}\right]\right\} \tag{1}$$

It is worthwhile to note that, although both the *Spectral Method* (in near-field Fresnel pattern calculation) and the *Direct Method* (in far-field Fresnel pattern calculation), discussed in details in the references below, adopted on performing the Fresnel transform are mathematically equivalent, the oscillatory behavior of the kernels makes them computationally different[1].

Thus discretization of Supplementary Eq. 1 will provide different application conditions from those in far-field Fresnel patterns calculation. A sampled version of Supplementary Eq. 1 is:

$$h(\mathbf{x}',\mathbf{p}) = \mathcal{F}^{-1}\left\{\mathcal{F}[U_i(\mathbf{x}',\mathbf{p})\Phi(\mathbf{x}')R(\mathbf{x}')] \times \exp\left[i2\pi\lambda f_{\mu lens}\sqrt{1-\left((m''\Delta f_x)^2+\left(n''\Delta f_y\right)^2\right)}\right]\right\} \tag{2}$$

where $\mathbf{x}' = \{m'\Delta x, n'\Delta y\}$, Δx and Δy are the sampling intervals in x and y direction in the input and output space, and m' and n' are integer sampling indices in the input space. Δf_x and Δf_y are the sampling intervals in Fourier space, where $\Delta f_x = \Delta f_y = \frac{1}{N\Delta x}$, and the Fourier coordinates $f_x = m''\Delta f_x$ and $f_y = n''\Delta f_y$. *N* is the sampling number of the point spread function (PSF) in lateral plane (x or y). m'' and n'' are integer sampling indices in Fourier space.

Admitting sampling just in the Nyquist limit[2], the range of distances can be calculated. The application condition is near-field Fresnel propagation in our case. The Nyquist condition discussed in the reference paper[1] can be written as:

$$f_{\mu lens} \leq \frac{N(\Delta x)^2}{\lambda} \tag{3}$$

In our model, the sampling number of the PSF is N = 151, and Δ*x* is the sampling interval on the microlens array plane, i.e. the pixel size. The propagation distance z equals the focal length of the lenslet ($f_{\mu lens}$ = 3 mm). The wavelength is λ = 532 nm in our case. Therefore, the condition for the sampling pixel size is given as

$$\Delta x \geq 3.25\ \mu\text{m} \tag{4}$$

In our case the sampling interval is chosen to be $10\ \mu\text{m}$ which satisfies the sampling condition.

References:

1. Mas, D. *et al.* Fast numerical calculation of Fresnel patterns in convergent systems. *Opt. Commun.* **227,** 245–258 (2003).

2. Mas, D., Garcia, J., Ferreira, C., Bernardo, L. M. & Marinho, F. Fast algorithms for free-space diffraction patterns calculation. *Opt. Commun.* **164,** 233–245 (1999).
